# Conditioned Canine Cadavers for Near-Natural Interprofessional Veterinary and Human Surgery Training

**DOI:** 10.7759/cureus.55049

**Published:** 2024-02-27

**Authors:** Thomas Eriksen, Jan Viberg Jepsen, Magnus Petur Bjarnason

**Affiliations:** 1 University Hospital of Companion Animals, University of Copenhagen, Copenhagen, DNK; 2 Copenhagen Academy for Medical Education and Simulation (CAMES), University of Copenhagen, Copenhagen, DNK

**Keywords:** low-fidelity models, general surgery residency training, surgery simulation, surgical careers, visceral surgery, ‘abdominal surgery’, interprofessional training, cadaver model

## Abstract

Both medical and veterinary students find that the use of cadavers is critical to learning anatomical structures and surgical techniques. The use of human cadavers and the resulting user emotions are driven by serious ethical issues that are currently much less pronounced in veterinary education. Ethically sourced canine cadavers, thus, are more readily available. Aesthetics such as odor and visual appearance, though, influence both learner and educator motivation. We have investigated a way of delaying cadaver decomposition by post-mortem in situ, chemical-free, gastrointestinal lavage. We are convinced that canine cadavers, conditioned as described here, will improve the outcome of cadaver-based surgical skills training by facilitating preparation, reducing the number of required cadavers, postponing decomposition, improving the surgeon's haptic-tactile response to organ and tissue handling and suturing, and, possibly most importantly, increasing learners' and educators' focus due to the significantly improved aesthetics. We hypothesize that skill transfer for medical students and doctors, because of the similar abdominal anatomy, may be easier when training with conditioned canine cadavers as compared to artificial simulators or pigs in vivo.

## Introduction

Varner et al. recently reviewed the use of cadavers in the education of veterinarians and medical doctors and the future perspectives of using cadavers [1]. The use of cadavers has primarily been in anatomy and surgical education. Both medical and veterinary students, as well as educators, are emotionally affected by the use of cadavers [2-5]. In medical education, the use of cadavers and the resulting emotions are driven by a range of ethical issues [3] that are currently much less pronounced in veterinary education [4]. Ethically sourced canine cadavers, thus, are more readily available. Both medical and veterinary students, though, are also emotionally affected by a range of aesthetic factors such as odor, visual appearance, and surrounding workspace environment [2-5]. Nevertheless, both medical and veterinary students find that the use of cadavers is critical to learning anatomical structures and surgical techniques [1,4].

Varner et al. also discuss cadaver preservation techniques. The most common are based on aldehydes, alcohol, and salt solutions. Aldehyde preservation, in particular, poses health risks and alters tissues' texture and consequently tissue haptic-tactile properties, dominated by rigidity. The use of fresh or fresh-frozen cadavers is not mentioned in the mini-review by Varner et al. [1]. We use canine and feline cadavers extensively in the surgical training of veterinary students. Not only in undergraduate clinical teaching but also in postgraduate continuing education. Unlike preserved cadavers, the workload in conditioning fresh cadavers is much less, and in fresh cadavers, haptic-tactile factors, except for bleeding tendencies and peristalsis, are nearly optimal. Health risks are minimal, provided the cadavers are free of zoonoses. However, fresh cadavers are very difficult to obtain in a plannable way, and scheduling teaching is, therefore, impossible. We use fresh-frozen canine cadavers, i.e., cadavers frozen immediately after euthanasia and thawed before use, for training in skin surgery, dental treatments, and orthopedics, including immobilization of limbs after joint surgery or osteosynthesis. Abdominal surgery, which constitutes a significant part of the surgical training curriculum, is very challenging in fresh-frozen cadavers [1]. Cadaverosis is, of course, somewhat inhibited during freezing, but the thawing period is long enough in larger cadavers that putrefaction and gas production increase rapidly during this phase [1,5]. This means that intestines, liver parenchyma, and bile ducts, and gall bladder in particular, change color and texture, often with autolysis and sloughing of intestinal walls, and always of intestinal mucosa. In addition, aesthetic factors, particularly odor, oral mucosal, and skin sloughing, are so unappealing that they affect students' focus and learning [2,5]. Consequently, the options for using fresh-frozen cadavers are not fully exploited, increasing the number of cadavers needed. Thoracic surgical procedures are less sensitive to cadaverous changes, and thoracoscopy and thoracotomy are possible, but they are still indirectly affected by the cadaverous changes in the abdomen.

Mathews et al. described a method in which the body is eviscerated, the lumen of the gastrointestinal tract is flushed free of feces and frozen separately in tap water, and the body cavities are flushed, filled with newspaper, and closed [5]. This removes the majority of bacteria causing tissue decomposition, and the eviscerated, fresh-frozen, and thawed body decomposes slower when kept refrigerated between uses and is workable for at least a couple of weeks. Organ sets also remain stable in the refrigerator, where cadaverous changes are minimal for at least one week, and they may be refrozen after use. The cadaver preparation is labor-intensive, and preparation of an animal typically takes an hour [5]. In addition, to achieve optimal surgical training output, an artificial body cavity must be used to hold the organ set, which prolongs preparation time and costs. We have used this method in postgraduate training, and the quality of the thawed body and organ sets is surprisingly good. The fat in the greater omentum tends to harden somewhat over time, but it is not a major problem except in procedures where omentalization is a central part of the procedure.

## Technical report

To preserve the organ-topographic relationship and postpone organ cadaverosis, we have investigated low-fidelity conditioning of canine cadavers with in situ, minimally invasive, and chemical-free, postmortem gastrointestinal lavage.

**Figure 1:**
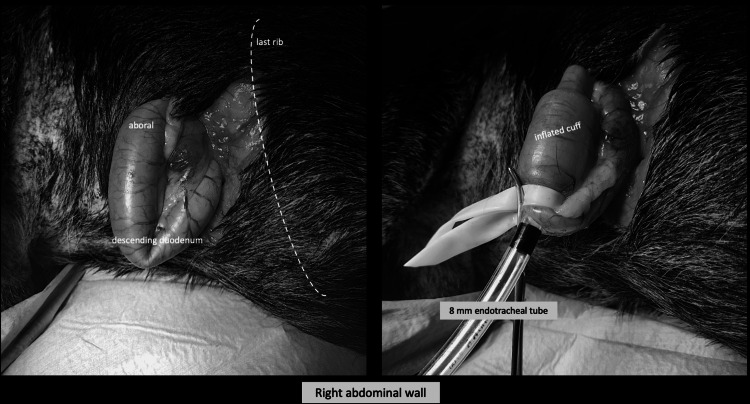
Right paracostal approach to duodenum Most oral descending duodenum exteriorized through small right side paracostal incision (left panel). Intraluminal position of 8 mm endotracheal tube with inflated cuff and Penrose drain tourniquet (right panel).

This method has proven to be faster and eliminates the need for artificial body cavities, leaving the organs with their natural topographic placement in intact body cavities. The applicability of this cadaver conditioning is technically simple and easy to learn (Figure 1), requires no specialized equipment, and follows these steps.

First, the animal is placed on its left side, and a small 5-10 cm paracostal laparotomy is performed on the right side, approximately 10 cm caudal to the last rib. The descending duodenum is palpated just aboral to the easily identifiable pylorus and brought gently forward into the incision. A Penrose drain or similar is placed around the most oral part of the duodenum. Immediately oral to the drain, a small incision is made on the antimesenteric side, and a tracheal tube of appropriate size (6 or 8 mm) is passed orally into the lumen of the intestine. The cuff is distended, and the Penrose drain is tightened to gently occlude the intestine and prevent backflow of the flushing fluid into the stomach. The cadaver is placed in an anti-Trendelenburg position, and the cuff connector is connected to a hose attached to a water tap. A standard-size garden hose is easily attached to the endotracheal tube connector. The intestines are flushed with a very gentle flow. Flushing continues until the fluid no longer contains feces. The abdomen may be manipulated gently to loosen any feces from the intestinal loops. When the intestine is empty, the tracheal tube is turned and passed orally through the pylorus into the pyloric antrum. The cuff is maximally distended, and the Penrose drain is gently tightened around the intestine. The largest possible gastric tube is inserted into the stomach. The cadaver is now placed in a Trendelenburg position, and the stomach is then flushed with a gentle flow. If the feed particles are large, it may be difficult to empty the stomach completely. Gentle manipulation of the cranial abdomen promotes emptying. When the stomach is completely empty, the gastric and tracheal tubes are removed, and the incision in the duodenum is closed with 3-4 staples. The abdominal wall is closed in an appropriate manner with sutures or staples. Finally, the fur coat is shaved, and the cadaver is frozen.

## Discussion

We use canine, feline, and porcine cadavers extensively for training both medical and veterinary students and postgraduates. Our observation is that nonconditioned cadavers decompose to an extent that makes them unusable for organ (thoracic/abdominal) surgery within 24 hours after thawing from a frozen state [5]. If the thawed cadavers are kept at refrigerator temperature (<5 °C), this period is slightly extended. During use at room temperature, the cadavers decompose quickly over four to six hours.

Porcine cadavers are widely used in the training of surgeons but cannot be conditioned following this protocol. This is mainly due to the ascending colon in pigs, which has a helical architecture with centripetal and centrifugal gyri assembled in a cone-like spiral. The ascending colon anatomy makes the abdominal topography of pigs very different from humans and dogs and demands maneuvers that are difficult and uncommon during canine and human abdominal surgery, and further, prevents effective lavage of the colon both ante- and retrograde [6,7]. However, we have used live anesthetized pigs for teaching canine surgery after removal of the ascending colon and side-to-side anastomosis of the ileum and transverse colon. This is somewhat time-consuming and leaves the abdomen with some signs after laparotomy. Following ascending colon ablation and ileo-colostomy, however, the overall abdominal topography will resemble both the canine and human abdominal topography to a very large extent. Unless the learning goals demand an intact abdomen or colon, it is our opinion that conditioning of porcine cadavers with the removal of the ascending colon and lavage of the remaining small and large bowel, as described here for canine cadavers, will delay decomposition correspondingly. If learning goals allow side-to-side anastomosis to be substituted with simple closure of the ileum and transverse colon ends, cadaver conditioning time will be significantly reduced.

Basic surgical skills such as tissue handling and suturing are easily trained in both canine and feline cadavers. However, the dermal-subdermal anatomy of dogs and cats is different from that in humans. Dogs and cats have thinner epidermis, and the skin is very movable due to a very loose subdermal connective tissue and panniculus. Training in skin advancement and axial pattern flaps, thus, is much easier in dogs and cats, which may be of benefit to novice trainees [1].

Open bowel anastomosis is easily trained in both canine and feline cadavers since GI anatomy and topography largely resemble the same in humans [6-8]. Bowel vascularity and serosal conditions are similar to human bowel, and the wide range of canine body sizes, in particular, may create an option for training open abdominal surgery in both adults and children. The radiating vascularity of the porcine small bowel is different from the arcuate vascularity in humans, dogs, and cats. Further, both dogs and cats have a very extended life-span expectancy, which makes them prone to lifestyle diseases such as obesity and cancers, while pigs at a weight of 25-35 kg are younger than four months and more correctly should be regarded as juvenile, comparable to infants between one and two years, and consequently more suitable as an animal model when teaching pediatric surgery Mikkelsen et al. [9].

Abdominal conditions, especially in large canine cadavers, are largely similar to the human abdominal cavity [6], which makes them suitable for training dexterity, laparoscopy with pneumoperitoneum, port placement, and triangulation, and to some extent for training maneuvers for surgical site presentation.

Canine cadavers are suitable for training trans- or retroperitoneal nephrectomy, adrenalectomy, endoscopic enteral anastomosis, gastric bypass, and cholecystectomy among other procedures [8].

Orthopedic procedures such as long bone fracture treatment with intramedullary pinning and plating, as well as corrections of growth deformities, are easily trained in both canine and feline cadavers. The hands and feet of the dog are less suitable for training surgery.

We have positive experience with using conditioned male canine cadavers for robotic prostatectomy. Female canine and feline uteri are bicornuate and thus quite different from the human uterus. The supporting structures of the uterus, however, such as the round, broad, and suspensory ligaments and vessels, are in principle similar.

The anatomy of abdominal organs and the abdominal topography of dogs and humans is not identical (Tables 1, 2).

**Table 1:** Comparative characteristics of abdominal organs in humans, pigs, and dogs [6,7].

	Kidney morphology comparable	Pancreas morphology comparable	Spleen morphology comparable	No. of distinct liver lobes and processes	Gall bladder
Human	Yes	Body and tail	Yes	5	Yes
Dog	Yes	Body, right and left lobe	Relatively large	7	Yes
Pig	Yes	Body and tail	Yes	6	Yes

**Table 2:** Comparative characteristics of the small and large intestines of humans, pigs, and dogs [6,7]. Characteristics of the small and large intestines of adult dogs vary greatly, depending on the breed.

	Small intestines (m)	Large intestines (cm)	Colon morphology	Vermiform appendix	Sigmoid flexure descending colon	Haustra colon caecum
Human	5-8	24	Inverted L-shape	Yes	Yes	Yes
Dog	2-5	69	Inverted L-shape	No	No	No
Pig	15-22	400	Cone shaped spiral	No	No	Yes

However, the topographical similarity and many macroscopic similarities of canine abdominal organs when compared to the pig may extend the use of conditioned canine cadavers to the surgical training of medical students and doctors as well, thus creating an option for an interprofessional approach to the teaching of surgery [8,10]. Our initial assessment of this interprofessional approach to surgery training of course needs further validation.

## Conclusions

After initial pilot experiments, we are convinced that this cadaver conditioning will optimize the use of the cadavers as a biological low-fidelity, near-natural surgical skills simulator. We are also convinced that it will improve teaching for veterinarians and veterinary students by facilitating preparation, reducing the number of required cadavers, postponing decomposition, improving the surgeon's haptic-tactile response to organ and tissue handling and suturing, and, most importantly, by increasing students' focus due to significantly improved aesthetics.
